# The Behaviour of Diphtheria in Schools

**Published:** 1913-02-01

**Authors:** 


### THE BEHAVIOUR OF DIPHTHERIA IN SCHOOLS.

This was the subject of discussion at the meeting of the Section of Epidemiology * on the 24th ult., presided over by Dr. Hamer. The paper was by Dr. J. A. H. Brincker, and he first dealt with the questions of notification, diagnosis, and control of the disease, especially injunctions to teachers, nurses, and others who come into contact with children to watch for suspicious illnesses and report them to the health authorities. Previous to the 'eighties, diphtheria was a disease of the rural population, but now it is essentially urban in incidence. London shows a distinct rise, even among towns, a fact well borne out by the reports and contributions of Sir Shirley Murphy, who demonstrated a special increase at the age period—three to ten. Dr. Downes found a greater mortality from the disease among females as compared with males—*i.e.*, in the proportion, for England and Wales, of 108 to 100. Sir Shirley Murphy showed in his report that the change in age incidence of mortality had its beginning about the time that compulsory school attendance had its beginning. Infection seems to be spread more particularly by cases of mild throat illness, which, though diphtheritic, show no other symptoms. All preventive schemes must recognise this very important fact. When investigating the course of diphtheria in schools, swabbing of the throats should be secondary to a careful inquiry, with the object of reconstructing the course taken by the outbreak. By that means it may be possible to discover the source and method of spread, whether by a convalescent carrier, a missed case, a precocious carrier, or a contact otherwise healthy. It is necessary to differentiate between true diphtheria bacilli and diphtheria germs; very few now believe in the potency of Hoffmann's bacillus. There is a marked difference in virulence between the various strains of the true diphtheria bacillus. Cobbett, in connection with an outbreak in Cambridge in 1900, examined 692 persons, and found 42 notified cases of diphtheria, and there were 22 other notified cases in the town. Cobbett stated that the principal means of combating diphtheria were, after the isolation of persons actually sick, the detection of those who, apparently in good health, went about carrying with them the diphtheria bacillus; and the isolation of these and of convalescents until the bacilli could no longer be cultivated from them. Porter, investigating an outbreak in 1911, attributed the spread of the infection to two chronic nasal carriers of long standing, an outbreak suppressed by vigorous swabbing and quarantine. C. J. Thomas, in "Report of School Medical Officer, 1904," based on a bacteriological survey of infected London schools, divided germ carriers into the following classes: 80 per cent. mild and unrecognised cases of diphtheria; 12 per cent. healthy contacts from infected houses; 6 per cent. cases of intermittent or recrudescent infection after discharge from hospital; 2 per cent. quite healthy cases. The majority of carriers are children between five and eight years.

For some years now there has been but little diphtheria in London, yet it continues to occur with seasonal regularity in certain parts and in certain schools in London. The disease has been for some time endemic in the better-housed, high-situated new parts of South-East London. Dr. Brincker said he was beginning to believe that the Mendelian law of inheritance of unit characters might be an influencing factor; and that another is the antagonism of germs. It was stated by Schiotz in 1910 that the *Staphylococcus aureus* would outgrow diphtheria germs; and diphtheria did not seem to occur amongst the dirtiest members of the community. The author then gave a most instructive account of outbreaks of the disease in six London schools, with tables of figures, and in conclusion emphasised, among other things, the great proportion of nasal carriers and the difficulty of impressing on teachers the importance of excluding all cases of suspicion and not readmitting them until a satisfactory investigation had been made.

* Royal Society of Medicine.

